# An Efficient Genotyping Method in Chicken Based on Genome Reducing and Sequencing

**DOI:** 10.1371/journal.pone.0137010

**Published:** 2015-08-27

**Authors:** Rongrong Liao, Zhen Wang, Qiang Chen, Yingying Tu, Zhenliang Chen, Qishan Wang, Changsuo Yang, Xiangzhe Zhang, Yuchun Pan

**Affiliations:** 1 School of Agriculture and Biology, Shanghai Jiao Tong University, Shanghai, China; 2 Faculty of Life Science and Technology, Kunming University of Science and Technology, Kunming, Yunnan, China; 3 National Poultry Engineering Research Center, Animal Husbandry and Veterinary Research Institute, Shanghai Academy of Agricultural Sciences, Shanghai, China; 4 Key Laboratory of Veterinary Biotechnology, Shanghai Jiao Tong University, Shanghai, China; Shenzhen Institutes of Advanced Technology, CHINA

## Abstract

Single nucleotide polymorphisms (SNPs) are essential for identifying the genetic mechanisms of complex traits. In the present study, we applied genotyping by genome reducing and sequencing (GGRS) method to construct a 252-plex sequencing library for SNP discovery and genotyping in chicken. The library was successfully sequenced on an Illumina HiSeq 2500 sequencer with a paired-end pattern; approximately 400 million raw reads were generated, and an average of approximately 1.4 million good reads per sample were generated. A total of 91,767 SNPs were identified after strict filtering, and all of the 252 samples and all of the chromosomes were well represented. Compared with the Illumina 60K chicken SNP chip data, approximately 34,131 more SNPs were identified using GGRS, and a higher SNP density was found using GGRS, which could be beneficial for downstream analysis. Using the GGRS method, more than 3528 samples can be sequenced simultaneously, and the cost is reduced to $18 per sample. To the best of our knowledge, this study describes the first report of such highly multiplexed sequencing in chicken, indicating potential applications for genome-wide association and genomic selection in chicken.

## Introduction

SNP chips have been widely used in genome-wide genetic marker genotyping in chicken [1–4]. However, the major limitations are that SNP genotyping data may be subject to an ascertainment bias due to the procedure used to select the SNPs [5,6], and the SNP chips that originated from special breeds that cannot be used to detect novel SNPs [7–9], particularly in indigenous chicken genetics research. For example, the commercially available Illumina 60K chicken SNP chip was constructed using only two meat-type chickens and two egg-type chickens [1], and the Affymetrix high density 600K SNP genotyping array employed twenty-four lines, including fifteen commercial lines, eight experimental inbred layers and one unselected layer line [10].

Recently, next-generation sequencing (NGS) technologies have been used in genome-wide genetic marker discovery and genotyping [11–14]. To date, three major reduced-representation sequencing methods employing restriction enzyme digestion of target genomes have been developed. Sequencing of reduced-representation libraries (RRLs) was first reported for cattle SNP discovery, and 66 cattle individuals from three populations constituted three libraries; as a result, 62,042 putative SNPs were identified and their allele frequencies were predicted [15]. Another method, restriction-site-associated DNA sequencing (RAD-Seq) was first used to sequence two model organisms; more than 13,000 SNPs were identified and three traits were mapped [16]. The third method is genotyping by sequencing (GBS), which was demonstrated with maize and barley recombinant inbred populations; and approximately 200,000 and 25,000 sequence tags were mapped, respectively [17]. These methods have largely improved the detection of genome-wide genetic markers; however, they all have some limitations. The RRL method can be well adapted to population genetic analysis and evolution; however, it does not distinguish samples because barcodes are not used. The RAD-Seq method is expensive and labor-intensive because of the complex procedures involved. Both RRL and RAD require microgram quantity genomic DNA to be used. The GBS method is a low-genome-coverage and low-sequencing-depth genotyping method that is suitable for marker discovery in highly inbred populations. However, GBS cannot be easily applied to general outbred populations (humans and some major farm animals) because of the high level of heterogeneity and phase ambiguity in the haplotypes.

Many reports have shown that NGS technologies are effective for chicken studies [11–14]. Although the genotyping costs have somewhat decreased with NGS development, the high costs have remained a barrier to its large scale application in chicken breeding. Our research group has developed a genotyping by genome reducing and sequencing (GGRS) method for outbreed pig populations [18]. Because the procedure is simple and highly reproducible for the reduction of genome complexity, GGRS is effectively applied. In this study, we applied GGRS for SNP discovery and genotyping in chicken with two modifications based on the properties of the chicken genome. A 252-plex GGRS library was constructed and successfully sequenced and demonstrated a simple and cost-effective genotyping method for chicken.

## Materials and Methods

### Animals and DNA samples

All of the experimental and animal procedures used in this study were strictly in accordance to the protocol approved by the Institutional Animal Care and Use Committee of Shanghai Jiao Tong University (Contract No. 2013–003303).

Two closed white leghorn lines, including 126 fast-feathering and 126 slow-feathering line chickens (Shanghai Xin Yang Poultry Breeding Center, Shanghai, China), were used. Blood samples from the brachial veins of the chickens were collected using standard venipuncture techniques. The genomic DNA from the blood was extracted using the standard phenol/chloroform method. The DNA quality was examined through agarose gel electrophoresis, and the DNA concentration was measured by spectrophotometry (TIAGEN, Beijing, China). High-quality and high-molecular-weight DNA was diluted in water to 50 ng/μL, as quantified using an intercalating dye (PicoGreen; Invitrogen, CA, USA).

### Preparation of the GGRS Sequencing Library

In this study, we improved two aspects of the GGRS protocol (http://klab.sjtu.edu.cn/GGRS/) [18] based on the properties of the chicken genome: 1) simplified the protocol and redesigned a set of 252 adapter-barcodes in a single lane to further reduce the cost, and 2) we replaced the common divergent “forked” adapter by using two different types of complementary adapters, which may help decrease the adapter dimers. The detailed protocols are as follows:

#### (1) Selection of restriction enzyme and fragment size

To sequence more samples in a single lane and to further reduce the cost, a selective methylation sensitive restriction enzyme (RE) was used to digest the samples and reduce the genome complexity. To determine the sample numbers for a lane, we first determined the total fragments to be sequenced per sample to ensure that sufficient genome-wide markers are used for the GWAS and GS. The total fragments were calculated by dividing the genome size by the extent of linkage disequilibrium (LD). The chicken LD varies greatly between different breeds and different chromosomes (GGA); some previous studies have reported that the LD ranged from 25 kb to 120 kb for commercial white layers [19] and was approximately 1 cM on GGA 10 and GGA 28 in an inbred Nutreco breed, whereas it was 15 kb on GGA 10 in an outbred Nutreco breed [20–22]. We chose 10 kb for the LD extent in this study to detect more markers and obtain a high physical resolution for studies; therefore, approximately 100,000 genomic locations can be sequenced. According to the RE *Ava*II *in silico* digestion results of the chicken reference genome (Gallus_gallus-4.0), more than 130,000 fragments between 200 bp to 400 bp were obtained; thus, *Ava*II was chosen for this study. The use of an Illumina HiSeq 2500 sequencer paired-end (2 x 100 bp) pattern (http://www.illumina.com/) can yield approximately 35 gigabase pairs of data per single lane. When 2% of the sample genome is sequenced with 5 x depth, a total of 257 samples can be pooled and sequenced in a lane, and considering the balance of base pairs, we constructed a 252-plex sequencing library herein.

#### (2) Design for adapters and barcodes

Two different types of adapters, namely the barcode adapter and the common adapter, were used [17]. Both the barcode adapter and the common adapter have an *Ava*II-compatible sticky end, and the barcode adapter has an “index” to identify the samples. In addition, the barcode adapter and the common adapter are both composed of top and bottom strands. The paired strands of each adapter were diluted in TE (10 mM Tris-Cl, pH 8.5) to 25 μM separately and annealed in a thermal cycler (ABI, USA) using the following cycling protocol: the temperature was maintained at 95°C for 2 min, decreased to 25°C by a rate of 0.1°C/s, maintained at 25°C for 30 min, and then hold at 4°C. The annealing barcode adapters and common adapters were then diluted in water to ~0.3 ng/μL. A total of 252 barcodes were generated by the GBS Barcode Generator (http://www.deenabio.com/services/gbs-adapters).

#### (3) Production of GGRS sequencing libraries

Each sample containing 100 ng of genomic DNA was digested with 5 U of *Ava*II (New England Biolabs, USA) in a volume of 15 μL at 37°C for 6 h, inactivated by heating at 80°C for 20 min and cooled to 4°C. Approximately 0.3 ng of the barcode adapter and 0.3 ng of the common adapter were then added to the digested sample DNA. Subsequently, a 20 μL volume ligation sample containing 400 cohesive end units of T4 DNA ligase (New England Biolabs, USA) was incubated at 22°C for 5 h and then inactivated at 65°C for 20 min. All of the ligation samples were then pooled.

The pooled samples containing all of the restriction fragments were directly used without any further modifications, and more than 5 μL of the pooled samples were amplified in 50 μL volumes containing 1x Premix Taq (Takara, Dalian, China) and 25 pmol of each of the following amplification primers:

5'-AATGATACGGCGACCACCGAGATCTACACTCTTTCCCTACACGACGCTCTTCCGATCT-3' and 5'-CAAGCAGAAGACGGCATACGAGATCGGTCTCGGCATTCCTGCTGAACCGCTCTTCCGATCT-3'. The amplification cycling protocol was as follows: one cycle of 72°C for 5 min and 94°C for 5 min, 16 cycles of 94°C for 30 s, 65°C for 30 s, and 72°C for 40 s, and a final extension step at 72°C for 10 min. At least eight amplification products were required to prepare a sequencing library. The amplification products were separated by electrophoresis on a 2% agarose gel, which allowed dimers removal and cleanup of the PCR products. Gel pieces containing DNA fragments between 300 bp to 500 bp (the 200-bp to 400-bp DNA fragments plus 100-bp adapters) were excised and then purified using a commercial kit (QIAquick Gel Extraction Kit, Qiagen, Germany) according to the manufacturer’s instructions with the exception that the gel was melted at room temperature (18–22°C) without isopropanol adding [23].

The purified products (the sequencing library) were analyzed on a 2% agarose gel to measure the fragment sizes (approximately 300 bp to 500 bp). The quality of the sequencing library was further evaluated using an Agilent 2100 bioanalyzer. To obtain a suitable sequencing library, the fragment peaks and the major fragments should both be in the range of 300 bp to 500 bp, and the adapter dimers (~ 128 bp in length) and primer dimers (~60 bp in length) should be either be at an minimal amount or absent (**Fig A in S1 File**). The suitable library was sequenced on a HiSeq 2500 sequencer with a paired-end (2 x 100 bp) pattern (Genewiz, Suzhou, China).

### Sequencing data processing and SNP identification

All of the sequencing reads generated from the HiSeq 2500 sequencer were within two groups, denoted R1 and R2. R1 included the reads with the barcode adapters, and R2 included the reads from the common adapter fragments. The NGS QC toolkit was used to evaluate the read quantity and quality [24]. The raw sequencing reads were filtered using the following criteria: (1) exactly matched one of the barcodes; (2) the four-base remnant of the *Ava*II cut site (GWCC) were after the barcode; (3) no adapter dimmers; and (4) no non-calling bases throughout all of the 100 bases. A unique read is recorded when one or more remaining reads (good reads) map to the same genome position. The distribution of unique reads was used to evaluate the sample representation and sequencing results.

The good reads were aligned to the chicken reference genome (Gallus_gallus-4.0) using the Burrows-Wheeler Aligner (BWA Version 0.7.12) with the default settings [25]. The alignment reads with mapping quality scores greater than 20 were then used to call SNPs with SAMtools (Version 0.1.19) [26]. The missing genotypes were imputed by iBLUP (http://klab.sjtu.edu.cn/iBLUP/) [27]. Genomic loci that were genotyped with calling quality scores greater than 20 (99% accuracy) in more than 63 (25%) samples and achieved an average sequencing depth greater than 5 x with an MAF greater than 0.05 were considered SNPs and were retained for further analyses.

## Results

### Read quantity and quality

Two different sequencing libraries were constructed using the two different types of complementary adapters and the common divergent “forked” adapters. The electropherogram results showed that the primer dimers and the adapter dimers were either rarely observed or absent in the PCR products of the library constructed using the complementary adapters, and no dimers were found in the sequencing library; however, the primer dimers and the adapter dimers were clearly observed in the PCR products and the adapter dimers were obviously found in the library constructed using the "forked" adapters (**S2 File**).

**Fig 1:**
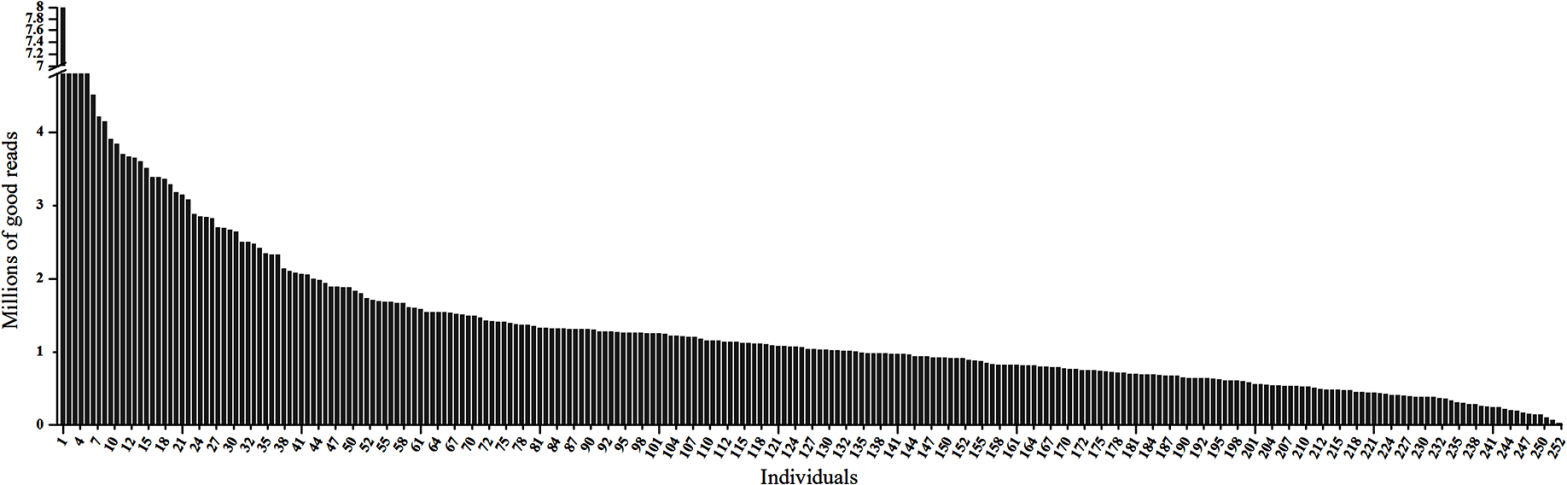
Distribution of the good reads in the 252 samples. The x-axis denotes the 252 sequencing samples, and the y-axis denotes the millions of good sequencing reads.

Of the total 398,262,858 raw sequence reads, 343,788,777 reads (86.3%) were good reads. Excluding two samples with 79,764 and 37,918 good reads, the maximum and minimum numbers of good reads were 8,386,592 and 113,070, respectively (**Fig 1**).

The average Phred quality scores (*Q*) [28,29] for both the R1 and R2 reads were greater than *Q*30 (with a base call accuracy of 99.9%) (**S1 Fig**). The average quality scores for each position with the exception of the second and last positions were greater than *Q*30 (**S1 Fig**).

The 252-plex chicken sequencing data have been submitted to the Short Read Archive of the National Center for Biotechnology Information (Accession number: SRX751211).

### Sample and SNP representation

**Fig 2:**
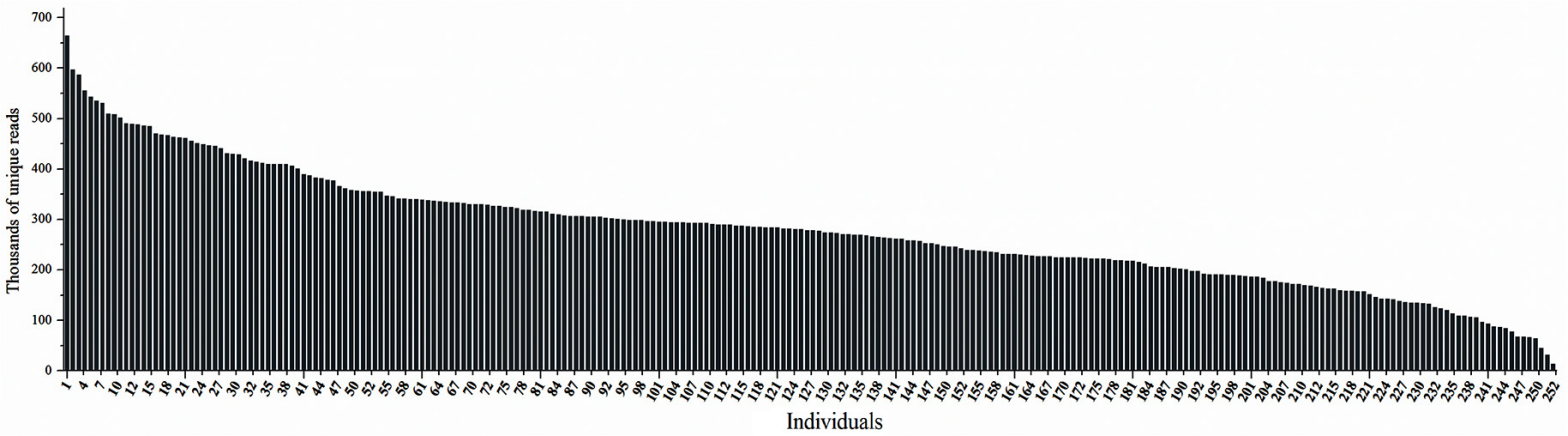
Distribution of the unique reads across the 252 samples. The x-axis denotes the 252 sequencing samples, and the y-axis denotes the thousands of unique reads.

The sequencing results showed that all of the 252 samples and all of the chromosomes that comprised the chicken reference genome were well represented. The average number of good reads was 1,364,241 reads per sample, the average sequencing coverage was 2.2% and the average depth per sample was approximately 5. The coefficient of variation (CV = standard deviation/mean) of the good reads was 69% after exclusion of the five maximal and two minimal samples. Moreover, compared with the good reads, the unique reads varied less between samples (**Fig 2**). The unique reads can reflect the overlapped sequencing regions, and the CV of the unique reads was at a low level of approximately 40%, which denoted that the sequencing regions largely overlapped across individuals.

**Fig 3:**
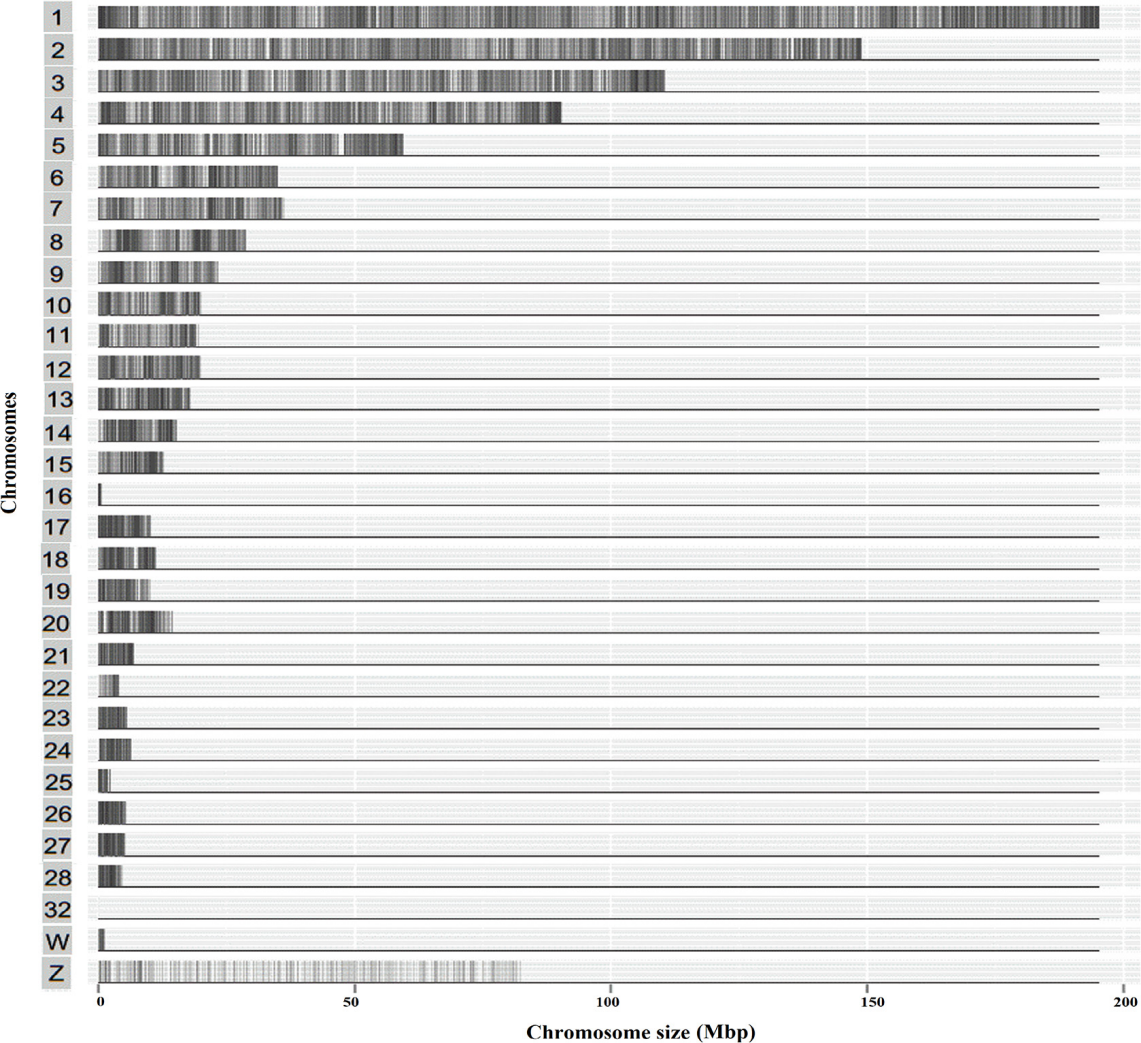
Distribution of the GGRS SNPs across the chromosomes. The x-axis denotes the chromosome size (Mbp), and the y-axis indicates the chromosomes. If at least one SNP was present in each 10-kb genome block, we draw at least one grey lines on the special chromosome positions. If no SNPs are found in a block, we used a white color. Therefore, the deeper the color, the higher the number of SNPs.

According to the criteria (see Methods), a total of 91,767 SNPs were ultimately identified. The SNPs appeared to be mostly evenly distributed across the chromosomes (**Fig 3**), but a higher density was observed toward the telomeres in some of the chromosomes, and the highest density biases were observed in the telomeres on GGA 1, 2, 4, 5 and 16. Because only 32 SNPs were found in GGA32, these are barely shown in Fig 3. The SNP density of heterosome Z (~15.61) was lower than that of the other chromosomes (~1197); thus, these SNPs were thinly distributed on the chromosome. Compared to the SNPs in chicken dbSNP (ftp://ftp.ncbi.nih.gov/snp/organisms/chicken_9031/VCF/), we found that 47.5% (43576) of GGRS SNPs in common with the known chicken SNP database.

### Comparison with SNP chip data

**Fig 4:**
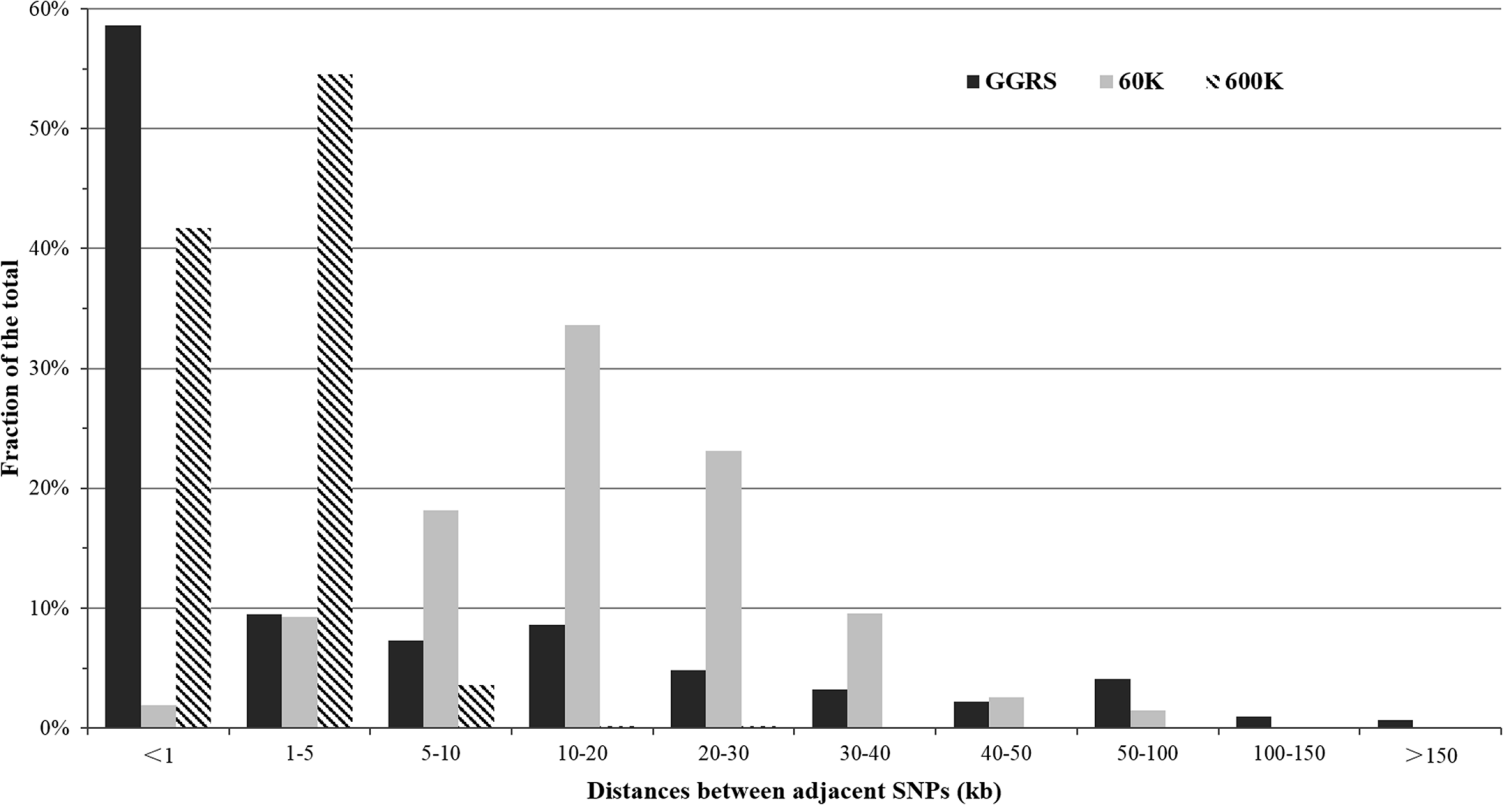
Distances between adjacent SNPs. Distribution of the distances between adjacent SNPs obtained using the GGRS, the 60K and 600K chicken SNP chips. The x-axis denotes the distances between adjacnt SNPs (kb), and the y-axis denotes the fraction of the total SNPs.

To evaluate the GGRS results, comparisons were performed among the GGRS SNPs and the commonly used SNP chip markers (the 60K and 600K chicken SNP chip data downloaded from http://www.animalgenome.org/repository/chicken/; the two SNP chips contained 57,636 and 580,954 markers, respectively) [1,10]. Large differences in the SNP numbers between the three methods were found for every chromosome existed (**Table 1**). The number of GGRS SNPs of the five macrochromosomes (GGA1-5) was approximately twofold greater than that in the 60K chip and approximately one fifth of that in the 600K chip. Moreover, the five macrochromosomes account for approximately 60% of the chicken genome and 47% of the total genes. The results showed that approximately 58%, 46% and 52% of the SNPs identified using the GGRS, the 60K and 600K chips were found across the five macrochromosomes, respectively (**Table 1**). The distribution of distances between adjacent SNPs showed that 75.4% of the GGRS SNPs tightly linked within 10 kb (LD extent) distances, 29.4% and 99.8% for the 60K and 600K chips, respectively (**Fig 4**). As shown in Table 1, the mean GGRS SNP density (total SNP number / chromosome length) was approximately 0.91 SNP per 10 kb, which was higher than that found for the 60K chip (0.56 SNP per 10 kb) but markedly lower than that obtained for the 600K chip (5.76 SNPs per 10 kb). The SNP numbers were depended on the genetic variabilities of the sequencing breeds, and the GGRS method focused on the sequencing reads. A total of 70,456,726 unique reads were generated, 7 Gb sequences were obtained from all unique reads, and 28 Mb sequences were obtained for a single individual, which were much more than the 60K (57,636) and 600K (580,954) chips. The gene density (total gene number / chromosome length) for each chromosome was also calculated. On average, the SNP density of the 60K chip was approximately three-fold greater than the gene density, whereas the GGRS SNP density was approximately six-fold higher than the gene density, and the SNP density of the 600K chip was thirty-fold greater than the gene density(**Table 1**).

**Table 1:** Number of genes and SNPs per chromosome obtained from the GGRS and the 60K and 600K SNP chips.

Chromosome		Numbers				Density (N/Mbp)			
Number^a^	Size(Mbp)	Genes	GGRS SNPs	60K SNPs	600K SNPs	Genes	GGRS SNPs	60K SNPs	600K SNPs
1	195.28	2798	19171	8517	102501	14.33	98.17	43.61	524.89
2	148.81	1926	12549	6460	64755	12.94	84.33	43.41	435.15
3	110.45	1549	8761	4900	57517	14.02	79.32	44.36	520.75
4	90.22	1475	7513	3966	43569	16.35	83.27	43.96	482.92
5	59.58	1191	5097	2598	30907	19.99	85.55	43.61	518.75
6	34.95	640	3230	2042	22055	18.31	92.42	58.43	631.04
7	36.24	653	2822	2134	21792	18.02	77.87	58.89	601.32
8	28.77	633	2814	1679	17334	22	97.81	58.36	602.5
9	23.44	532	2114	1403	18236	22.7	90.19	59.85	777.99
10	19.91	515	1888	1570	19046	25.87	94.83	78.85	956.6
11	19.4	442	1461	1519	14021	22.78	75.31	78.3	722.73
12	19.9	407	2095	1614	14918	20.45	105.28	81.11	749.65
13	17.76	441	2383	1385	11373	24.83	134.18	77.98	640.37
14	15.16	487	2259	1197	13246	32.12	149.01	78.96	873.75
15	12.66	430	1377	1235	10542	33.97	108.77	97.55	832.7
16	0.53527	86	491	29	602	160.67	917.29	54.18	1124.67
17	10.45	376	1939	1023	9494	35.98	185.55	97.89	908.52
18	11.22	377	1653	1077	10063	33.6	147.33	95.99	896.88
19	9.98	383	1213	993	9123	38.38	121.54	99.5	914.13
20	14.3	443	1840	1840	9786	30.98	128.67	128.67	684.34
21	6.8	286	1057	902	8988	42.06	155.44	132.65	1321.76
22	4.08	147	407	463	4727	36.03	99.75	113.48	1158.58
23	5.72	268	861	751	6734	46.85	150.52	131.29	1177.27
24	6.32	214	937	869	7820	33.86	148.26	137.5	1237.34
25	2.19	255	579	235	2690	116.44	264.38	107.31	1228.31
26	5.33	279	1276	815	6446	52.35	239.4	152.91	1209.38
27	5.21	334	1296	590	5804	64.11	248.75	113.24	1114.01
28	4.74	299	948	777	5589	63.08	200	163.92	1179.11
Z	82.36	1146	1286	3010	26038	13.91	15.61	36.55	316.15
Total	1001	19012	91317	55593^b^	577187	37.48	154.4	86.6	576.17

The chromosome size and number of genes were obtained from the chicken assembly Gallus_gallus-4.0 (http://www.ncbi.nlm.nih.gov/genome/111).

^a^To ensure consistency across the three methods, without considering GGA32, heterosome W, LinkageGroups and unmapped contigs, only the other autosomes and heterosome Z are listed.

^b^Without considering the SNPs on the LinkageGroups and unmapped contigs.

## Discussion

To efficiently discover high-density genome-wide markers, we improved two main aspects of the GGRS protocol: 1) we simplified the protocol and redesigned a set of 252 adapter-barcodes in a single lane to further reduce the cost, and 2) we replaced the common divergent "forked" adapter using two different types of complementary adapters, which may help decrease the adapter dimers. In this study, a 252-plex GGRS library was constructed and successfully sequenced. All of the 252 samples were well represented with the exception of a few samples with extremely high or low numbers of good reads. We speculated that the read numbers obtained for samples may be due to poor DNA quality [30], accurate quantification of the high-molecular-weight DNA [17] and/or contamination of the DNAs with phenol/chloroform [31]. Additionally, the few high read numbers were mostly due to accurate quantification of the high-molecular-weight DNA, which remains a procedural bottleneck in multiplexed sequencing protocols [17]. Subsequently, the CV was 69% for the 252-plex library obtained in this study, which was somewhat higher than those obtain for the 72-plex pig GGRS library (CV = 43%) [18] and the 48-plex GBS library (CV = 43%) [17]; but lower than that obtained for the 96-plex *Drosophila* multiplexed shotgun genotyping library (CV = 89%) [32]. With regard to the highly multiplexed sequencing library, the CV obtained in this study was acceptable. To date, no studies have performed such highly multiplexed sequencing in chicken or even other animals.

Strict data filtering rules and SNP identification criteria were used to ensure high SNP confidence. For example, only reads with no non-calling bases throughout all 100 bases were retained, whereas some other studies retained reads with no non-calling bases in the first 72 [17] or 80 bases [18]. In addition, the loci that were genotyped with calling quality scores of more than 20 in more than 63 (25%) samples and an average sequencing depth greater than 5 x with an MAF higher than 0.05 were considered SNPs and were retained for further analysis. The iBLUP imputation method imputes missing genotypes using both identity-by-descent and linkage disequilibrium information; therefore, iBLUP imputes missing genotypes with greater accuracy than other imputation methods, such as BEAGLE. Even at a high missing rate of 70%, iBLUP can retain a high accuracy of 0.95, which is lower than that retained by BEAGLE (0.82) [27].

The chicken genome size is approximately 35% that of mammalian genomes but contains similar gene numbers, which illustrates that the chicken genome is a compact genome [33]. Compared with the 60K chicken SNP chip, the GGRS method generated 34,131 more SNPs, and the identified SNPs were distributed evenly across chromosomes. However, the 600K SNP chip contains 580,954 SNPs and has the highest density among the three methods. The GGRS method is an open and flexible method that can detect novel SNPs with different design, whereas the chips with fixed SNPs cannot. When the sequencing coverage increasing to 10%, then the resulting SNPs number will be equivalent to that of the 600K chip. In chicken, GGA16 possesses only 535-kb reference sequences but contains the highly variable major histocompatibility complex (MHC) [34,35]. A total of 491 SNPs were identified in GGA16 using GGRS, which is obviously higher than that (29 SNPs) identified using the 60K chicken SNP chip but somewhat lower than that obtained using the 600K SNP chip. The SNP density of heterosome Z was the lowest, which may be due to its lower gene density than that of the autosomes, and previous studies have reported a lower recombination rate on Z than on autosomes and an approximately four-fold reduction in diversity in heterosome Z [36,37].

Approximately 100,000 chicken markers can be detected in a short time using the GGRS method with a cost of $4,500 (USD) (approximately $18 per sample). Furthermore, using the GGRS method outlined here, 3528 samples (252 samples per channel, seven channels per flow cell, and two flow cells per run) can be sequenced simultaneously. The Illumina HiSeq SBS Kit V4 and the HiSeq cluster kits V4 (http://products.illumina.com/products/hiseq-sbs-kit-v4.html) with improved cluster density and throughput were recently released; and these allow the generation of up to 1 terabase (Tb) of data in a six-day run. Therefore, by employing the new Illumina kits, approximately 7056 samples (504 samples per channel, seven channels per flow cell and two flow cells per run) can be sequenced simultaneously, and the cost will be reduced to $10 or less per sample in the future. Additionally, with improvements in data processing, GGRS can also be applied in copy number variation (CNV) studies in the future. In conclusion, the GGRS method is an efficient and cost-effective genotyping method for chicken and it may be an alternative option for chicken breeding and research.

## Supporting Information

S1 Fig Average base quality scores of the reads. The average base quality scores of the R1 and R2 reads were calculated using the NGS QC toolkit.
(TIF)

S1 File Distribution of the fragment size in the suitable and unsuitable GGRS libraries. The library was run on an Agilent 2100 bioanalyzer. The x-axis denotes the fragment size (bp), and the y-axis denotes the fluorescence units (FUs, which indicate the concentration). The standard size peaks are at 35 bp and 10,380 bp. The suitable GGRS library. The fragment peak and major fragments in the suitable GGRS library are in the range of 300 bp to 500 bp, and this library has no adapter dimers or other dimer peaks **(Fig A)**. The unsuitable GGRS library **(Fig B)**.
(TIF)

S2 File Electropherograms of the libraries constructed using two different types of complementary adapters and the single “forked” adapters. PCR products of the fragments with complementary adapters. The primer dimers are clear, but the adapter dimers were either found rarely or absent **(Fig A)**. The PCR products of the fragments with single “forked” adapters. The primer dimers and the adapter dimers are clearly indicated **(Fig B)**. The purified PCR products (the sequencing library) from **Fig A (Fig C)**. The purified PCR products from **Fig B**. The adapter dimers are clearly indicated **(Fig D)**.
(TIF)
