# Supplementary figures and images for: An Efficient Genotyping Method in Chicken Based on Genome Reducing and Sequencing
Source: PLoS One. 2015 Aug 27;10(8):e0137010. doi: 10.1371/journal.pone.0137010 (PMC4551734; doi:10.1371/journal.pone.0137010)

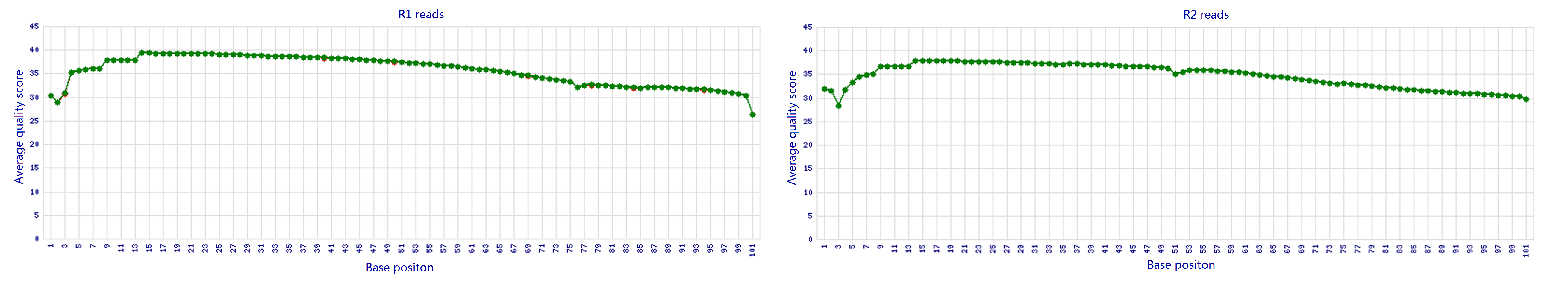

Supplement: S1 Fig — The average base quality scores of the R1 and R2 reads were calculated using the NGS QC toolkit. (TIF) [file pone.0137010.s001.tif]

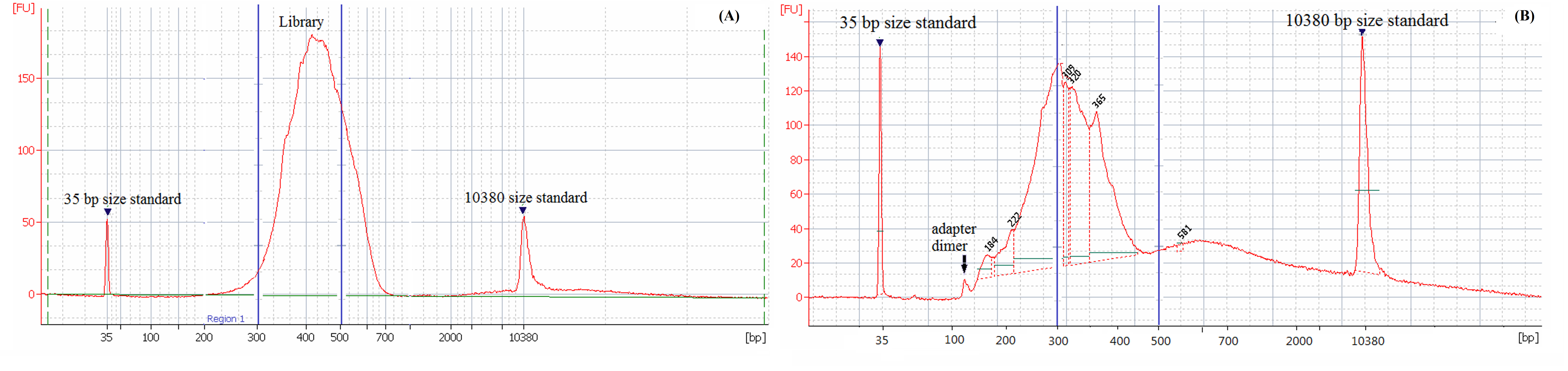

Supplement: S1 File — The library was run on an Agilent 2100 bioanalyzer. The x-axis denotes the fragment size (bp), and the y-axis denotes the fluorescence units (FUs, which indicate the concentration). The standard size peaks are at 35 bp and 10,380 bp. The suitable GGRS library. The fragment peak and major fragments in the suitable GGRS library are in the range of 300 bp to 500 bp, and this library has no adapter dimers or other dimer peaks (Fig A). The unsuitable GGRS library (Fig B). (TIF) [file pone.0137010.s002.tif]

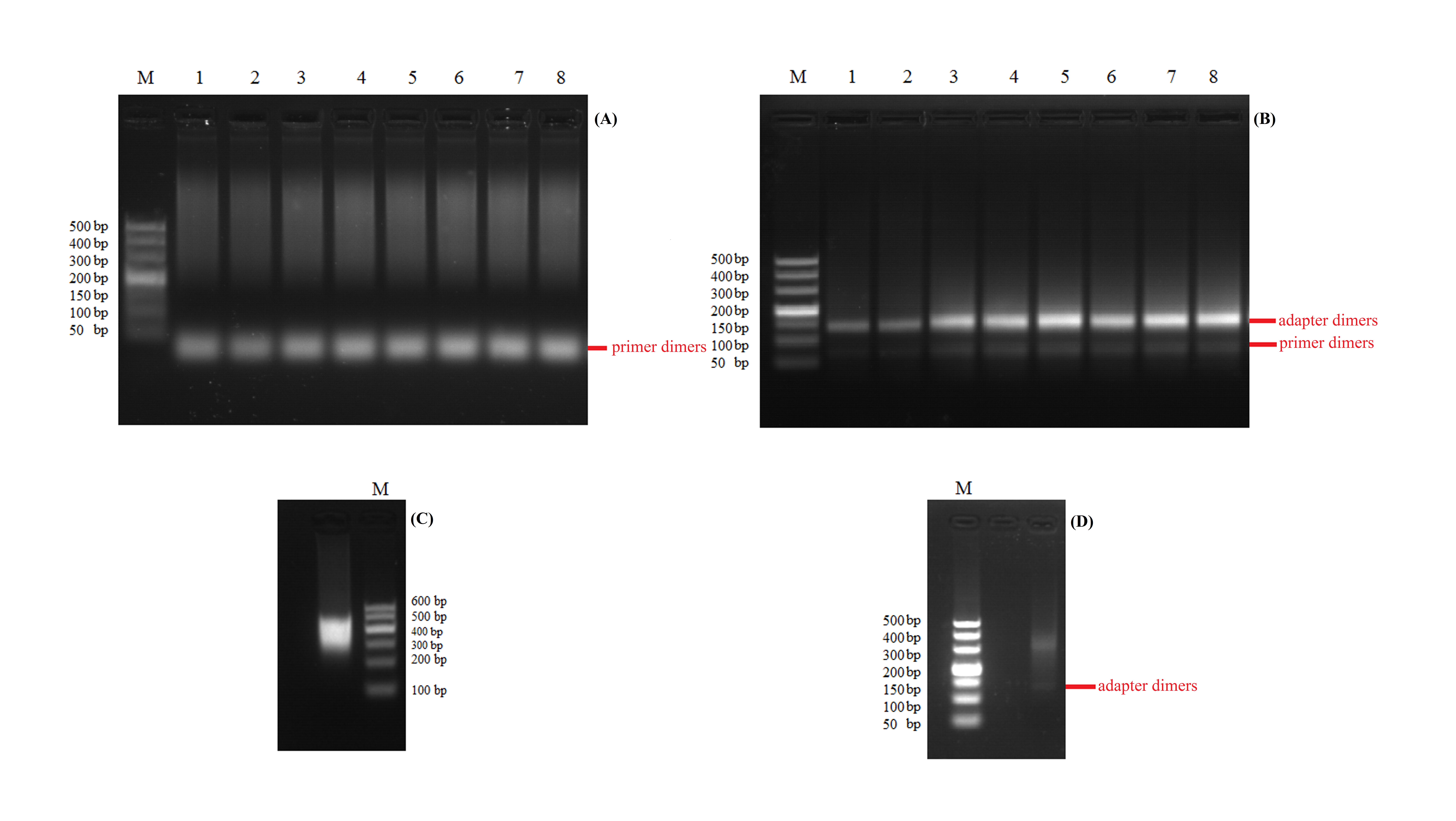

Supplement: S2 File — PCR products of the fragments with complementary adapters. The primer dimers are clear, but the adapter dimers were either found rarely or absent (Fig A). The PCR products of the fragments with single “forked” adapters. The primer dimers and the adapter dimers are clearly indicated (Fig B). The purified PCR products (the sequencing library) from Fig A (Fig C). The purified PCR products from Fig B. The adapter dimers are clearly indicated (Fig D). (TIF) [file pone.0137010.s003.tif]
